# Development of a Novel Short Synthetic Antibacterial Peptide Derived from the Swallowtail Butterfly *Papilio xuthus* Larvae

**DOI:** 10.4014/jmb.2003.03009

**Published:** 2020-07-15

**Authors:** Seong Ryul Kim, Kwang-Ho Choi, Kee-Young Kim, Hye-Yong Kwon, Seung-Won Park

**Affiliations:** 1 Sericultural and Apicultural Materials Division, National Academy of Agricultural Science, RDA, Wanju 55365, Republic of Korea; 2 Department of Biomedical Science, Daegu Catholic University, Gyeongsan 38430, Republic of Korea

**Keywords:** Antimicrobial peptide, hybrid peptide, papiliocin, Jelleine, biological safety

## Abstract

Insects possess biological defense systems that can effectively combat the invasion of external microorganisms and viruses, thereby supporting their survival in diverse environments. Antimicrobial peptides (AMPs) represent a fast-acting weapon against invading pathogens, including various bacterial or fungal strains. A 37-residue antimicrobial peptide, papiliocin, derived from the swallowtail butterfly *Papilio xuthus* larvae, showed significant antimicrobial activities against several human pathogenic bacterial and fungal strains. Jelleines, isolated as novel antibacterial peptides from the Royal Jelly (RJ) of bees, exhibit broad-spectrum protection against microbial infections. In this study, we developed a novel antimicrobial peptide, PAJE (RWKIFKKPFKISIHL-NH_2_), which is a hybrid peptide prepared by combining 1–7 amino acid residues (RWKIFKK-NH_2_) of papiliocin and 1–8 amino acid residues (PFKISIHL-NH_2_) of Jelleine-1 to alter length, charge distribution, net charge, volume, amphipaticity, and improve bacterial membrane interactions. This novel peptide exhibited increased hydrophobicity and net positive charge for binding effectively to the negatively charged membrane. PAJE demonstrated antimicrobial activity against both gram-negative and gram-positive bacteria, with very low toxicity to eukaryotic cells and an inexpensive process of synthesis. Collectively, these findings suggest that this novel peptide possesses great potential as an antimicrobial agent.

Several antibiotics are often used, sometimes in large amounts, for the treatment of infectious diseases in humans and farm animals [12]. However, most strains of pathogens have developed antibiotic resistance due to their use, and potential abuse [9, 12]. Antimicrobial peptides (AMPs) are biologically active molecules frequently used for the treatment of pathogenic bacteria [9]. Most AMPs are produced by numerous organisms, as an essential component of their innate immune response [13]. AMPs may be applied to treat infections caused by various microbes, even drug-resistant strains, because they have distinctive antimicrobial mechanisms compared with traditional antibiotics [9]. Thus, the development of new, natural antimicrobial peptides, with bacterial cell selectivity (high antimicrobial activity without cytotoxicity), may be applied to treat the infections caused by drug-resistant microbes [9, 12, 13].

Insects possess biological defense systems, such as passive structural barriers and cellular and humoral immune responses that can effectively combat the invasion of external microorganisms and viruses, thereby supporting their survival in diverse environments [4]. In other words, unlike the acquired immune factors in higher animals, the immune system of insects effectively protects them by secreting innate immune factors.

Insects primarily deploy AMPs to protect themselves against invading pathogens including bacteria, fungi, and viruses [5, 19]. They are produced rapidly in the fat bodies and other specific tissues of insects after sepsis/injury or an immune challenge, and subsequently released into the hemolymph to act against potentially pathogenic microbes [2].

Papiliocin, a 37-residue cecropin-like antimicrobial peptide derived from the swallowtail butterfly *Papilio xuthus* larvae, has been reported to demonstrate significant antimicrobial activity against several human pathogenic bacterial and fungal strains [3, 5, 19]. In insects, the cecropins constitutes of a large family of cationichelical AMPs that are active against gram-positive and gram-negative bacteria. Our previous report showed that the N-terminal helix of papiliocin played an important role in interacting with the bacterial cell membrane as well as with the lipopolysaccharides (LPS), which activated the immune system [5]. These results implied that strong interaction between papiliocin and LPS may affect the disruption of LPS structures, potentially leading to its high anti-inflammatory activity and the susceptibility of gram-negative bacteria to papiliocin. Fontana *et al.* had isolated a novel antibacterial peptide, Jelleine, from the Royal Jelly (RJ) of bees and reported its properties [1]. Jelleines are very short peptides, consisting of 8 to 9 amino acid residues, produced constitutively to provide broad-spectrum protection against microbial infections [1]. The Jelleines present a hypothetical net charge of 1+ or 2+, and most of these residues are hydrophobic [1]. Therefore, Jelleines represent the basic structural properties of the antimicrobial peptides.

It is essential to study the properties of peptides to develop new antimicrobial agents for both, gram-negative and gram-positive bacteria. AMPs play essential roles in peptide interactions with the membrane surface and/or the membrane core. Thus, the design of potent peptides have to take into consideration factors such as length, charge distribution, net charge, volume, amphipaticity, and oligomeric state in solution [1]. In this study, we designed and synthesized a novel hybrid peptide, PAJE (RWKIFKKPFKISIHL-NH_2_), composed of 1–7 N- terminal amino acid residues of papiliocin (RWKIFKK-NH_2_) followed by 1–8 N-terminal residues of Jelleine-1 (PFKISIHL-NH_2_) to develop a potent antibacterial peptide with a broad-spectrum of activity against gram- positive and gram-negative bacteria. N-terminal helix residues of papiliocin are essential for the rapid permeabilization of the gram-negative bacterial membrane [6]. Our results suggest that a novel 15-mer non-toxic antimicrobial peptide is highly effective against gram-negative and gram-positive bacteria.

PAJE (a 15-mer peptide), Jelleine-1 (8-mer peptide), and melittin (26-mer peptide) were synthesized in an automated solid-phase peptide synthesizer at a peptide synthesis facility, ANYGEN Co. (Korea) (Table 1). The synthetic peptides were purified by reverse-phase high performance liquid chromatography using Shimadzu C18 analytical (5 mm, 0.46 cm × 25 cm) and preparative C18 (10 mm, 2.5 cm × 25 cm) columns. Chromatographic separations were conducted using a water-acetonitrile linear gradient (0–80% of acetonitrile) containing 0.1% (v/v) trifluroacetic acid. The correct identity of the synthetic peptides was verified by matrix-assisted laser desorption ionization time-of-flight mass spectrometer (AXIMA Assurance, Shimadzu, Japan). The concentration of peptides was quantified using UV spectrophotometer.

Antibacterial activities of the peptides were determined against both gram-positive and gram-negative bacterial strains, including *Escherichia coli* (KACC 1039), *Klebsiella pneumoniae* (KCTC 2242), *Pseudomonas aeruginosa* (KACC 10259), *Staphylococcus aureus* (KACC 10196), *Bacillus subtilis* (KACC 19623), and *Enterococcus faecalis* (KACC 11304) (Table 2). Antifungal activities were monitored with the *Candida albicans* (KACC 30071) fungal strain (Table 2).

The antibacterial activity of synthetic PAJE was examined by the agar well diffusion assay. Briefly, single colonies of bacteria were inoculated in tryptic soy broth (TSB; Difco, USA) and cultured overnight at 37°C. The cultures were subsequently diluted in fresh TSB and incubated until the optical density at 600 nm reached 0.4. A volume containing 4 × 10^6^ CFU bacteria was inoculated into 10 ml of worm citrate phosphate buffer (9 mM sodium phosphate, 1 mM sodium citrate, pH 7.4) containing 1% low electroendosmosis-type agarose (Sigma- Aldrich, USA) and 0.03% TSB. The mixture was rapidly poured into a sterile petri-dish to form a uniform layer, after which 3-mm-diameter holes were punched in the set agarose and filled with 10 μl of test peptides. After allowing 3 h for diffusion of the samples, 10 ml of TSB medium containing 1% agar was overlaid and incubated overnight at 37°C. Activity of the synthetic peptide was measured from its inhibitory zone.

In addition to the agar well diffusion assay, the MIC (minimum inhibitory concentration) of synthetic PAJE was also determined using the broth microdilution assay and compared with those of Jelleine-1 and melittin. Briefly, bacteria were grown overnight at 37°C and shaken at 200 rpm in TSB. The cultures were then washed twice with autoclaved 10 mM sodium phosphate buffer (pH 7.4), and re-suspended in fresh TSB to a final concentration of 2 × 10s^4^ colony forming units (CFU)/ml. Two-fold serial dilutions of peptides were prepared in 0.01% acetic acid. The diluted peptides (10 μl) were distributed in each well of 96-well microtiter plates and inoculated with 90 μl of bacterial suspension (2 × 10s^4^ CFU/ml) in TSB. After 18-h incubation at 37°C, growth inhibition was determined by measuring absorbance at 600 nm with a microplate reader. The lowest concentration of peptide required for the prevention of bacterial growth was defined as MIC. MIC values were calculated from the average of triplicate measurements.

The hemolytic activity of synthetic PAJE was assessed against human red blood cells (hRBCs). Jelleine-1 and melittin were used as positive control peptides. Erythrocytes were washed thrice with phosphate-buffered saline (PBS) (10 mM PBS, 150 mM NaCl, pH 7.4), and centrifuged at 1,000 ×*g* and 4°C for 10 min. A 100-µl cell suspension, diluted with 10 mM PBS (final concentration approximately 8%), was mixed with 100-µl of varying peptide stock solutions in 96-well microtiter plates. The resulting suspension was incubated for 1 h at 37°C. After a 5-min centrifugation at 1,000 ×*g* and 4°C, release of hemoglobin was monitored by measuring absorbance of the supernatant at 405 nm. The values for 0% and 100% hemolysis were determined using erythrocyte suspensions incubated in 10 mM PBS and 0.1% Triton X-100, respectively.

Time-kill kinetic analysis of PAJE was performed for *E. coli* (KACC 1039) and *S. aureus* (KACC 10196), as described previously by Klepser *et al*. [7]. Bacterial suspensions (2 × 10^6^ CFU/ml) were incubated with 2× MIC at 37°C. Viability counts were performed at specific intervals (0, 10, 20, 40, 60, 90, 120, 180, and 240 min), and all experiments were performed at least twice. Melittin was used as a positive control peptide. Dose-dependent bactericidal activity of PAJE and melittin was measured against *E. coli* (KACC 1039) and *S. aureus* (KACC 10196). The bacterial suspensions (2 × 10^6^ CFU/ml), containing either PAJE or melittin (1, 2, 4, 8, 16, 32 μg/ml), were incubated at 37°C for 1 h. Aliquots (100-µl) were serially diluted in PBS and plated on NA medium. Colonies were counted after 18-h incubation at 37°C. Three independent experiments were performed.

Recently, a large number of AMPs have been identified and characterized from a variety of insects [5]. Papiliocin was reported to have significant antimicrobial activities against both gram-positive and gram-negative bacterial strains and the fungal strain *C. albicans* [5, 6, 8, 14, 15]. Furthermore, Jelleine-1 was found to be active against most bacterial species (gram-positive and gram-negative) as well as the yeast examined [1].

In this study, we developed a novel hybrid PAJE peptide as an AMP (Fig. 1). PAJE contained the 1–7 amino acids (RWKIFKK) of the N-terminal spiral structure of papiliocin, isolated from the larva of tiger butterfly, and 1–8 amino acids (PFKISIHL) of Jelleine-1 isolated from Royal Jelly, to form a reduced form of positive electrode and hydrophobic amino acid residues. The final amino acid sequence of PAJE was RWKIFKKPFKISIHL-NH_2_, a 15- mer peptide, which consisted of hydrophobic (red circle) and hydrophilic (blue circle) amino acids. The net charge and hydrophobicity (%) of the peptide sequence are shown in Table 1. The hydrophobic face facilitates its penetration into the membrane, thereby disturbing the bilayer curvature [10, 11, 14, 16, 18]. Higher hydrophobicity has frequently been observed to cause higher antimicrobial activity [14]. The efficacy of AMPs has also been observed to vary with the net positive charge, which enhances its binding to the negatively charged membrane by electrostatic effect [14, 15].

PAJE, a synthetic peptide, demonstrated antimicrobial activity against *E. coli* (gram-negative bacteria) and *S. aureus* (gram-positive bacteria), as observed from the agar well diffusion assay (Fig. 2). The antimicrobial activity of PAJE was higher than that of Jelleine-1 (160 μg/ml) used as a control. However, PAJE showed antibacterial activity against *E. coli* and *S. aureus* even at low concentrations (20–40 μg/ml).

The MIC values are summarized in Table 2. As expected, PAJE proved to be active against all microorganisms tested, with MIC values of 1–4 μM. It evidenced profound growth-inhibitory effects on gram-negative bacteria (MIC value of 1.0 μM). These effects of PAJE were stronger than those of melittin (four times), papiliocin (four times), and Jelleine-1 (eight times). One of the most desired properties of AMPs is its low toxicity to eukaryotic cells. To study the cytotoxicity of the newly designed peptide, we determined its ability to lyse hRBC. PAJE showed no hemolytic activity against hRBC, even at high concentrations (Table 3), suggesting that it is not detrimental to eukaryotic cells.

Sterilization rate analysis of PAJE against *E. coli* and *S. aureus* was performed using a time-kill curve assay (Fig. 3). PAJE showed very high antibacterial activity and time-dependent bactericidal activity; it had a similar microbicidal effect as melittin. Furthermore, we added different concentrations of PAJE to check dose- dependence of its activity (Fig. 4). The peptide was found to inhibit *E. coli* growth by approximately 99.9% after 1 h, at a minimum concentration of 16 μg/ml, and *S. aureus* growth by approximately 50% after 1 h at a treatment concentration of 16–32 μg/ml (Fig. 4).

In conclusion, we report the development of a novel 15-mer antimicrobial peptide that was highly effective against both gram-negative and gram-positive bacteria, the former being more prone. It exhibited a higher net charge than Jelleine-1, and was as efficient as melittin as a potent antimicrobial peptide. The hydrophobicity (%) of PAJE was higher than that of melittin. Therefore, our synthesized peptide exhibited a lower MIC (1 µM), than melittin, against the four tested microbes. This study suggests that the novel 15-mer short peptide is a highly effective antimicrobial agent. It exhibited no cytotoxicity and its synthesis was relatively inexpensive, due to its small size. The new peptide possesses great potential as an antimicrobial agent.

## Figures and Tables

**Fig. 1:**
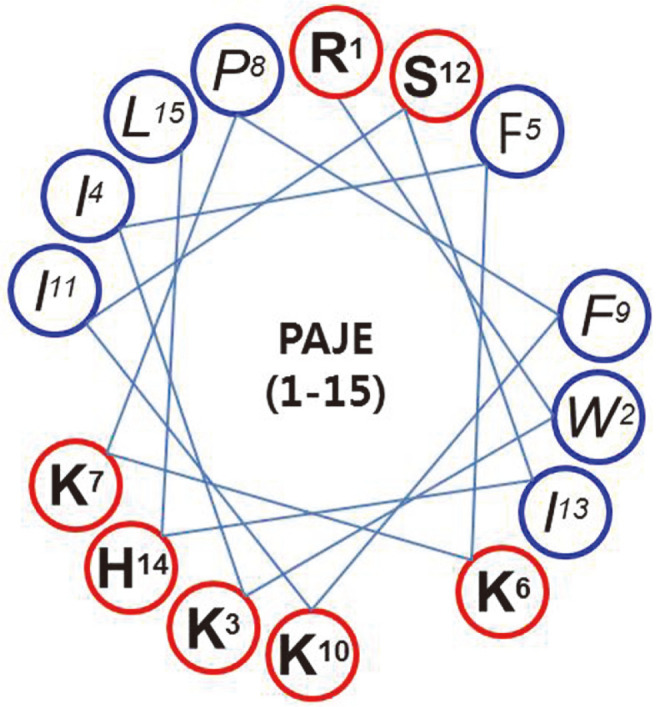
Helical wheel diagram of PAJE. The hydrophobic residues are indicated in red circle and hydrophilic residues are shown in blue circle.

**Fig. 2:**
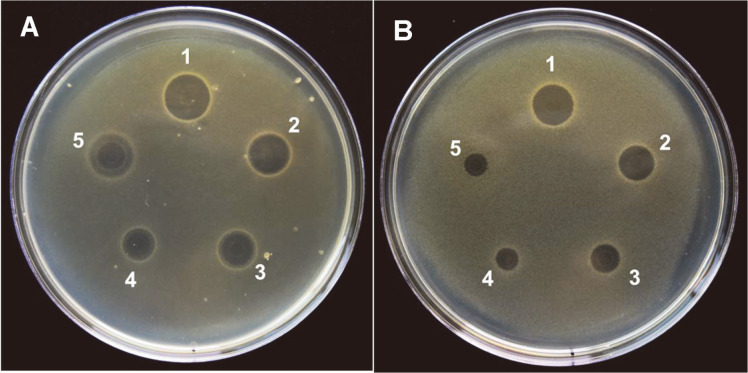
Measurement of antimicrobial activity. The antibacterial diffusion assay performed using the agar plates of PAJE against *E. coli* KACC 1039 (**A**) and *S. aureus* KACC 10196 (**B**). Position (1) PAJE 160 μg/ml; position (2) PAJE 80 μg/ml; position (3) PAJE 40 μg/ml; position (4) PAJE 20 μg/ml; and position (5) Jelleine-1 160 μg/ml.

**Fig. 3:**
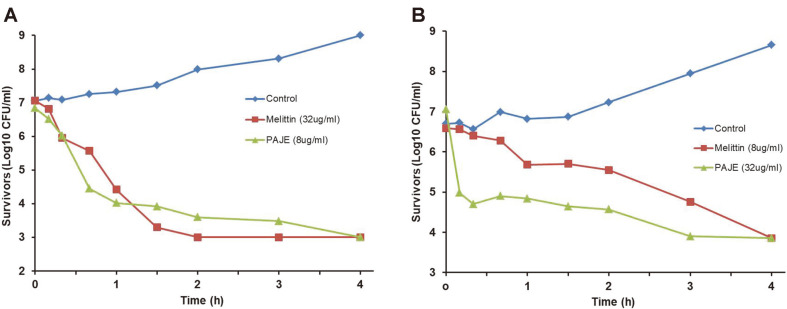
Results of time-kill curve assay. Time-kill kinetics of PAJE against *E. coli* and *S. aureus*. (**A**) Time-kill curves for 8 μg/ml of PAJE and 32 μg/ml of melittin in *E. coli*. (**B**) Time-kill curves for 32 μg/ml of PAJE and 8 μg/ml of melittin in *S. aureus*. All experiments were performed at least twice.

**Fig. 4:**
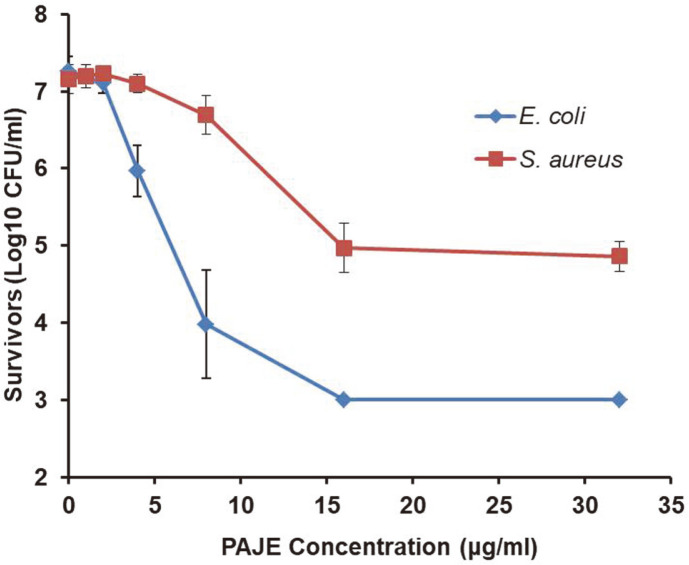
Dose-dependent antimicrobial activity. Bactericidal activities of PAJE against *E. coli* and *S. aureus* were measured after 1-h incubation at different concentrations. All experiments were performed in triplicate. The error bars indicate standard deviations from the mean.

**Table 1:** Amino acid sequences and properties of the peptides.

Peptides	Amino acid Sequences	Net charge	Hydrophobicity (%)	Molecular mas	

				Calculated	Observed
Jelleine-1	PFKISIHL-NH_2_	+1	50	954.2	952.8
PAJE	RWKIFKKPFKISIHL-NH_2_	+5	46	1941.4	1939.5
Papiliocin	RWKIFKKIEKVGRNVRDGIIKAGPAVAVV	+7	35	4002.8	4003.4
	GQAATVVK-NH_2_				
Melittin	GIGAVLKVLTTGLPALISWIKRKRQQ-NH_2_	+5	38	2847.5	2845.8

**Table 2:** Antimicrobial activity of PAJE.

Microorganisms	Minimum Inhibitory Concentration (MIC, μM)			

	Jelleine-1	Papiliocin	PAJE	Melittin
Gram-negative bacteria				
*E. coli* (KACC 1039)	8	4	1	4
*K. pneumonia* (KCTC 2242)	8	1	1	4
*P. aeruginosa* (KACC 10259)	8	1	1	4
Gram-positive bacteria				
*S. aureus* (KACC 10196)	16	8	4	1.5
*B. subtilis* (KACC 10196)	4	8	1	1.5
*E. faecalis* (KACC 11304)	16	8	4	2
Yeast fungi				
*C. albicans* (KACC 30071)	16	10	4	2

**Table 3:** Hemolytic activity of PAJE.

Peptides	% Hemoysis (μM)					

	50	12.5	6.25	3.125	1.56	0.78
PAJE	0	0	0	0	0	0
Melittin	100	100	100	95	93	31

## References

[1] Fontana R, Mendes MA, de Souza BM, Konno K, César LM, Malaspina O (2004). Jelleines: a family of antimicrobial peptides from the Royal Jelly of honybees (*Apis mellifera*). Peptides.

[2] Hoffman JA, Kafatos FC, Janeway CA, Ezekowitz RA (1999). Phylogenetic perspectives in innate immunity. Science.

[3] Hwang B, Hwang, JS, Lee J, Kim JK, Kim SR, Kim Y (2011). Induction of yeast apoptosis by an antimicrobial peptide, Papiliocin. Biochem. Biophys. Res. Commun..

[4] Hwang JS, Lee J, Hwang B, Nam SH, Yun EY, Kim SR (2010). Isolation and characterization of Psacotheasin, a novel Knottin-type antimicrobial peptide, from *Psacothea hilaris*. J. Microbiol Biotechnol..

[5] Kim SR, Hong MY, Park SW, Choi KH, Yun EY, Goo TW (2010). Characterization and cDNA cloning of a cecropin-like antimicrobial peptide, papiliocin, from the swallowtail butterfly, *Papilio Xuthus*. Mol. Cells.

[6] Kim J, Jacob B, Jang M, Kwak C, Lee Y, Son K (2019). Development of a novel Short 12-meric papiliocin-derived peptide that is effective against gram-negative sepsis. Sci. Rep..

[7] Klepserv ME, Malone D, Lewis RE, Ernst EJ, Pfaller MA (2000). Evaluation of voriconazole pharmacodynamics using time-kill methodology. Antimicrob. Agents Chemother..

[8] Lee J, Hwang JS, Hwang B, Kim JK, Kim SR, Kim Y (2010). Membrane perturbation induced by papiliocin peptide, derived from Papilio xuthus, in Candida albicans. J. Microbiol. Biotechnol..

[9] Lei J, Sun L, Huang S, Zhu C, Li P, He J (2019). The antimicrobial peptides and their potential clinical applications. Am. J. Trans. Res..

[10] Leontiadou H, Mark AE, Marrink SJ (2006). Antimicrobial peptides in action. J. Am. Chem. Soc..

[11] Melo MN, Ferre R, Castanho MA (2009). Antimicrobial peptides: linking partition, activity and high membrane-bound concentrations. Nat. Rev. Microbiol..

[12] Nikaido H (2010). Multidrug resistance in bacteria. Annu. Rev. Biochem..

[13] Pushpanathan M, Gunasekaran P, Rajendhran J (2013). Antimicrobial peptides: versatile biological properties. Int. J. Pept..

[14] Qi X, Zhou C, Li P, Xu W, Cao Y, Ling H (2010). Novel short antibacterial and antifungal peptides with low cytotoxicity: efficacy and action mechanisms. Biochem. Biophys. Res. Commun..

[15] Raguse TL, Porter EA, Weisblum B, Gellman SH (2002). Structure-activity studies of 14-helical antimicrobial beta-peptides: probing the relationship between conformational stability and antimicrobial potency. J. Am. Chem. Soc..

[16] Rathinakumar R, Wimley WC (2008). Biomolecular engineering by combinatorial design and high-throughput screening: small, soluble peptides that permeabilize membranes. J. Am. Chem. Soc..

[17] Son K, Kim J, Jang M, Chauhan AK, Kim Y (2019). Effects of C-terminal residues of 12-mer peptides on antibacterial efficacy and mechanism. J. Microbiol. Biotechnol..

[18] Zelezetsky I, Tossi A (2006). Alpha-helical antimicrobial peptides-using a sequence template to guide structure-activity relationship studies. Biochim. Biophys. Acta.

[19] Zhang L, Gallo RL (2016). Antimicrobial peptides. Curr. Biol..

